# Feasibility and Safety of a Ketogenic Diet During Systemic Therapy for Metastatic Renal Cell Carcinoma: Results from the Cetorein Pilot Study

**DOI:** 10.3390/nu18111712

**Published:** 2026-05-27

**Authors:** Cyrielle Rolley, Merzouka Zidane, Cosmina Nedelcu, Magalie Barth, Patrick Saulnier, Vincent Procaccio, Pierre Bigot

**Affiliations:** 1 Department of Urology, Angers University Hospital, 49333 Angers, France; pibigot@chu-angers.fr; 2 Department of Pathology, Angers University Hospital, 49333 Angers, France; 3 Department of Radiology, Angers University Hospital, 49333 Angers, France; 4 Department of Genetics, Angers University Hospital, 49333 Angers, Franceviprocaccio@chu-angers.fr (V.P.); 5 Department of Statistics, Angers University Hospital, 49333 Angers, France

**Keywords:** ketogenic diet, renal cell carcinoma, immunotherapy

## Abstract

Background: Metastatic renal cell carcinoma (mRCC) remains incurable despite advances with immune checkpoint inhibitors and tyrosine kinase inhibitors. Metabolic interventions, such as the ketogenic diet (KD), may modulate tumor biology and systemic inflammation, yet clinical evidence in mRCC is limited. Objective: To evaluate the feasibility, safety, and tolerability of KD combined with systemic therapy in mRCC patients. Design, Setting, and Participants: CETOREIN was a non-randomized, single-center pilot study enrolling 21 adult mRCC patients initiating systemic therapy. KD was initiated concurrently with treatment for up to 12 months, with follow-up at 1, 3, 6, and 12 months. Intervention: Participants followed a 2:1 KD (≈80% fat, 20% protein + carbohydrates) with dietitian-led counseling, medium-chain triglyceride supplementation, food diaries, and ketonuria monitoring. Outcome Measurements and Statistical Analysis: The primary endpoint was feasibility, defined by diet-related adverse events. Secondary endpoints included adherence, metabolic parameters, and exploratory clinical outcomes (response rate, progression-free survival [PFS], overall survival [OS]). All efficacy-related outcomes were descriptive and exploratory only. Results: Eight patients (40%) completed 12 months on KD, with a mean duration of 7 months. Common diet-related toxicities were diarrhea (55%), weight loss (45%), hypercholesterolemia (40%), and dyspepsia (30%), with no severe events. Early weight loss was modest and transient. Ketonuria correlated with dietary records, confirming adherence. Median PFS was 9.5 months, and median OS was 39 months. Among four patients undergoing cytoreductive nephrectomy, exploratory paired PD-L1 analyses showed decreased expression in three cases; however, these observations are hypothesis-generating only and cannot be attributed to the ketogenic diet. Conclusions: KD is feasible and demonstrated an acceptable tolerability profile in selected mRCC patients, though long-term adherence is challenging. No conclusions regarding antitumor efficacy can be drawn from this small non-randomized pilot study. Future studies should evaluate shorter interventions and optimized dietary protocols in larger randomized trials.

## 1. Introduction

Renal cell carcinoma (RCC) represents a major global health burden. In 2022, approximately 430,000 new cases were diagnosed worldwide, resulting in 155,000 deaths [1]. At diagnosis, 10–20% of patients have synchronous metastases, and an additional 10–30% will develop metachronous metastatic disease following nephrectomy for localized tumors. Current first-line systemic therapies combine immune checkpoint inhibitors (ICI) and targeted agents [2].

Clear-cell RCC is characterized by profound metabolic and inflammatory remodeling. Tumor progression occurs within a chronic inflammatory renal niche where angiogenic, metabolic, and immune pathways interact synergistically to reshape the tumor immune microenvironment [3]. This inflammatory context plays a key role in oncogenesis and tumor maintenance. At the metabolic level, RCC cells display impaired mitochondrial oxidative phosphorylation and enhanced aerobic glycolysis, consistent with the “Warburg effect” [4]. Transcriptomic data from The Cancer Genome Atlas confirm the downregulation of oxidative phosphorylation pathways and upregulation of glycolytic genes in clear-cell RCC [5]. In vivo metabolic tracing using [U-13C] glucose in patients undergoing nephrectomy further demonstrated increased glycolysis and reduced glucose oxidation in tumor tissue compared to normal kidney tissue [6].

There is strong patient interest in metabolic or dietary interventions. A recent survey of 289 individuals treated for metastatic RCC found that 34.9% of patients modified their diet for potential therapeutic benefit, and 18.8% adopted a ketogenic diet despite limited evidence of its efficacy or safety in this setting [7]. Dietary interventions may also influence systemic inflammation: for example, ketogenic metabolic profiles have been associated with reduced inflammatory markers in patients with prostate cancer under active surveillance [8]. Furthermore, the ketogenic diet (KD) may support kidney function in specific populations, such as individuals with obesity or autosomal dominant polycystic kidney disease [9]. While these effects should require further investigation, they could be relevant in metastatic RCC, where chronic kidney disease is common due to tumor-related renal destruction or prior nephrectomy.

The ketogenic diet is a high-fat, very low-carbohydrate regimen that induces hepatic production of ketone bodies such as acetoacetate, β-hydroxybutyrate, and acetone, serving as alternative energetic substrates to glucose. Initially developed for refractory epilepsy [10], KD has since been explored in obesity, type 2 diabetes, endocrine disorders, neurodegenerative diseases, and cancer [11,12]. Several biological mechanisms may confer potential anticancer effects: metabolic stress due to glucose restriction [13], reduction in circulating IGF-1 [14], inability of tumor cells with mitochondrial dysfunction to efficiently metabolize ketone bodies [15], inhibition of angiogenesis [16], and reduction in systemic inflammation [17]. Ketone bodies, particularly β-hydroxybutyrate, may also influence inflammatory signaling and immune modulation through immunometabolic pathways [18].

Preclinical findings in RCC further support the rationale for exploring KD in this disease. In vitro studies demonstrate that exposure to ketone bodies reduces proliferation and viability in RCC cell lines (ACHN and Renca cells) while increasing mitochondrial metabolic activity. In vivo, KD slowed tumor growth in mice and upregulated PD-L1 expression, enhancing responses to anti–PD-L1 monoclonal antibody therapy [19].

KD is a well-established therapeutic approach in drug-resistant epilepsy, with established protocols for initiation and monitoring. While generally safe, adverse effects include dehydration (47%), gastrointestinal symptoms (40%), hyperuricemia (36%), micronutrient deficiencies, dyslipidemia (27%), and kidney stones (3%), among others [20]. By contrast, ICI therapy carries a distinct profile of immune-related adverse events affecting different organs, including dermatologic, gastrointestinal, pulmonary, endocrine, musculoskeletal, and renal toxicities [21]. Understanding the combined safety of KD and systemic immunotherapy in metastatic RCC is therefore of clinical relevance.

Given the metabolic dependencies of RCC, the role of inflammation within its tumor microenvironment, coupled with the strong interest of patients in dietary interventions, and promising preclinical data supporting synergy between KD and immunotherapy, we initiated the CETOREIN study. This study aims to evaluate the safety and tolerability of a 2:1 ketogenic diet in patients receiving systemic therapy for metastatic RCC.

## 2. Materials and Methods

### 2.1. Ethics Statement

CETOREIN was a single-center, non-randomized pilot study conducted in the Department of Urology at Angers University Hospital. The protocol was approved by an ethics committee and registered on ClinicalTrials.gov (NCT04316520) on 20 March 2020. All participants provided written informed consent prior to enrollment.

### 2.2. Study Design and Participants

The study enrolled adult patients (≥18 years) with metastatic renal cell carcinoma requiring systemic therapy. Eligibility criteria comprised measurable disease according to RECIST v1.1, Eastern Cooperative Oncology Group (ECOG) performance status ≤1, and adequate biological parameters. Major exclusion criteria included contraindications to the ketogenic diet or to systemic therapy, including uncontrolled gastrointestinal disorders, inability to maintain oral intake, or clinical situations considered incompatible with safe ketogenic intervention by the nutrition and oncology teams.

**Figure 1:**
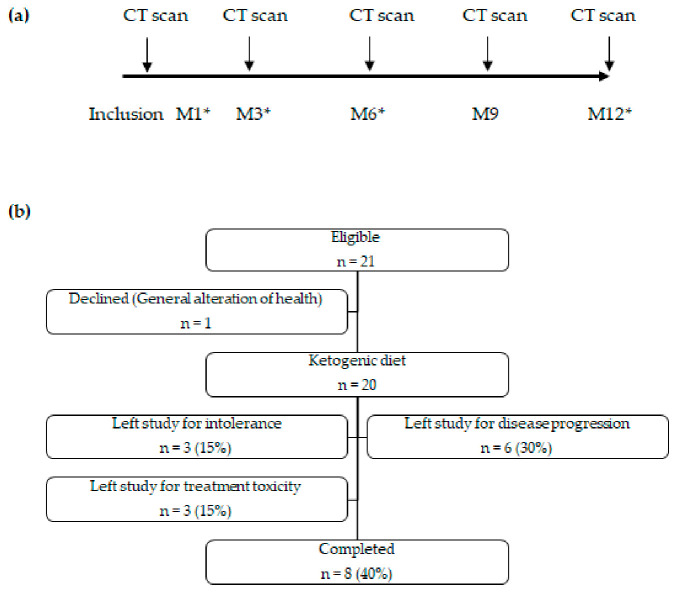
Study design and diagram of inclusions. (**a**) Study design and course of treatment. * Dietary, clinical, and laboratory evaluations. (**b**) Flowchart showing the recruitment process of the study.

The ketogenic diet (KD) was initiated concurrently with the start of systemic treatment. Patients were assessed at 1, 3, 6, and 12 months following KD initiation (Figure 1). Each follow-up visit included dietary interviews, vital signs, laboratory assessments, adverse-event reporting, and medication review. Radiological evaluation using CT scans was conducted every three months. PD-L1 expression was analyzed in patients who subsequently underwent cytoreductive nephrectomy after KD initiation.

### 2.3. Intervention: Ketogenic Diet Procedures

Patients were prescribed a ketogenic diet with an initial target ratio of 2:1 (fat to combined protein and carbohydrates), corresponding approximately to 80% fat and 20% protein plus carbohydrates, for 12 months. Prior to initiation, each participant received standardized education from the nutrition team regarding the general principles of the ketogenic diet and practical cooking instructions.

A dietician provided individualized counseling with specific caloric and protein targets, adapted according to body weight, nutritional status, and tolerance to systemic treatment. In obese or nutritionally vulnerable patients, lower ketogenic ratios could be implemented to preserve adequate protein intake and maintain nutritional status. Dietary counseling was also adjusted in cases of anorexia, diarrhea, or significant weight loss. Consequently, the 2:1 ratio should be considered a therapeutic target rather than a ratio consistently maintained throughout follow-up.

Patients received daily micronutrient supplementation with FruitiVits^®^ (one sachet/day; Vitaflo International Ltd., Liverpool, UK) and medium-chain triglyceride supplementation with Betaquick^®^ (225 mL bottles; Vitaflo International Ltd., Liverpool, UK), provided by the study sponsor. Patients completed prospective food diaries, which were reviewed by dieticians to estimate total caloric intake and macronutrient distribution (carbohydrates, protein, and fat). Urinary ketones were monitored at home every two weeks using dipsticks.

### 2.4. Safety Assessment

Adverse-event attribution to the ketogenic diet versus systemic anticancer therapy was assessed jointly by the oncology and nutrition teams according to clinical judgment, temporal relationship with dietary intervention, response to dietary modifications, and consistency with the known toxicity profiles of both systemic treatments and ketogenic diets.

### 2.5. Systemic Treatments and Oncologic Evaluation

Oncological treatment was determined independently of the study protocol during multidisciplinary tumor board meetings, according to standard clinical practice, prognostic factors, patient performance status, and tumor histology. Disease status was evaluated by CT scan every three months according to RECIST v1.1. KD was discontinued in cases of disease progression or KD-related adverse events.

### 2.6. Endpoints

The primary endpoint was the feasibility of the ketogenic diet, defined as the frequency and severity of diet-related adverse events. Secondary endpoints included the proportion of patients adherent to the diet, progression-free survival, and overall survival. Clinical outcomes were collected as exploratory descriptive endpoints only.

### 2.7. Statistical Analysis

Data analyses were performed using IBM SPSS Statistics version 15.0. Given the pilot design and limited sample size, all analyses were exploratory and not powered for formal hypothesis testing. Continuous variables are presented descriptively as mean ± standard deviation. Normality assumptions were assessed before paired comparisons were explored descriptively. No adjustment for multiple comparisons was applied; therefore, *p*-values should be interpreted cautiously. Survival outcomes were analyzed descriptively using Kaplan–Meier estimates, and differences were assessed using the log-rank tests and Cox proportional hazards analyses.

## 3. Results

### 3.1. Baseline Characteristics

Twenty-one patients were enrolled between July 2020 and January 2022. One patient discontinued participation before effective ketogenic diet initiation; therefore, 20 patients were evaluable for dietary and safety analyses. Denominators are reported for each analysis and time point throughout the manuscript. Baseline characteristics and systemic treatments are summarized in Table 1. All patients had histologically confirmed renal cell carcinoma. Eighteen patients (85.7%) received first-line systemic therapy, and three (14.3%) were treated in later lines. According to Heng’s criteria, five patients (23.8%) had favorable-risk disease, 10 (47.6%) had intermediate-risk disease, and six (28.6%) had poor-risk metastatic RCC.

### 3.2. Feasibility

Eight patients (40%) completed the 12-month KD. Six patients discontinued KD due to toxicity: four discontinued because of gastrointestinal intolerance or poor dietary tolerability, two because of systemic treatment-related toxicity. Six patients experienced disease progression: four discontinued KD, whereas two elected to continue the diet outside the study protocol (Figure 1).

The mean duration of KD was 7 months (SD 2.4). Mean macronutrient distribution was 74% fat (SD 0.09), 17% protein (SD 0.03), and 9% carbohydrates (SD 0.02) (Table 2). Although the planned ketogenic ratio was 2:1, many patients required adjustment toward a lower ratio (approximately 1.5:1) to maintain adequate protein intake and improve tolerability.

**Figure 2:**
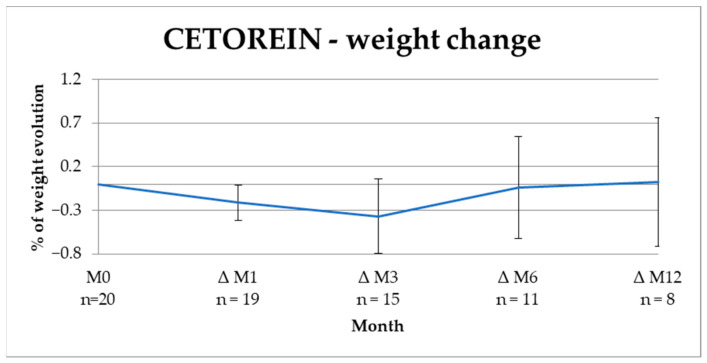
Weight change during ketogenic diet—CETOREIN study.

A modest early weight loss was observed during the first month after KD initiation, followed by subsequent stabilization or partial regain over time (Figure 2). Laboratory monitoring showed moderate increases in cholesterol and triglyceride levels during follow-up, whereas albumin, glucose, vitamin D, magnesium, selenium, and carnitine levels remained globally stable throughout the study period (Table 3).

**Figure 3:**
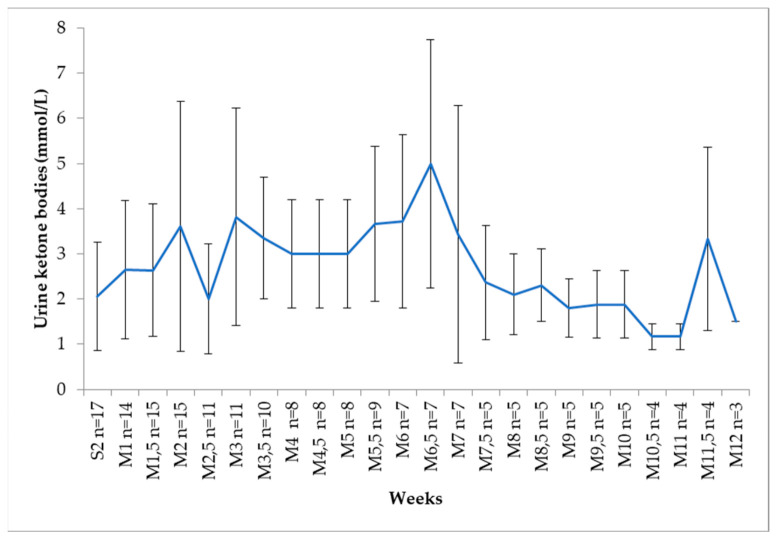
Mean urine ketone bodies evolution on urinary dipstick during ketogenic diet—CETOREIN study.

Ketosis was documented at least once in 16 patients. Urinary ketones were monitored using semi-quantitative dipstick measurements performed at home every two weeks. Home urine ketone monitoring was available in 14 patients; among them, six maintained stable ketosis (>1 mmol/L) (Figure 3).

### 3.3. Safety and Tolerability

No serious adverse events were attributed to the ketogenic diet. The most frequent KD-related adverse events were diarrhea (*n* = 11, 55%), weight loss (*n* = 9, 45%), hypercholesterolemia (*n* = 8, 40%), hypertriglyceridemia (*n* = 7, 35%), dyspepsia (*n* = 6, 30%), hyperuricemia (*n* = 4, 20%), hypoalbuminemia (*n* = 3, 15%), abdominal pain (*n* = 3, 15%), and anorexia (*n* = 2, 10%). All adverse events attributed to KD are summarized in Table 4. Laboratory parameter changes are detailed in Table 3.

### 3.4. Efficacy

After a mean follow-up of 35 months, seven patients (33%) had died from their disease. The median time to progression on KD was 11.5 months, including 9.5 months for patients receiving first-line therapy and 8.0 months for those treated in later lines.

**Figure 4:**
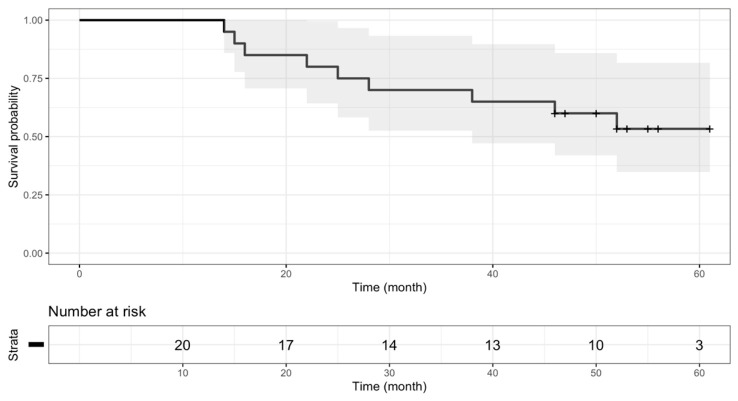
Overall survival—CETOREIN study.

**Figure 5:**
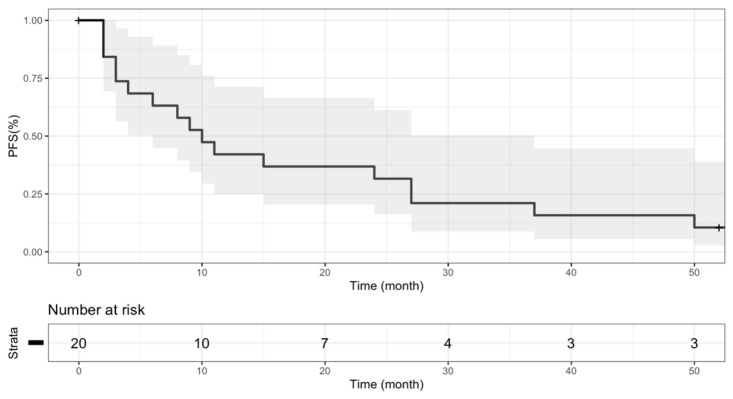
Progression-free survival—CETOREIN study.

Best treatment responses during the study period included three complete responses (15%), three partial responses (15%), nine stable diseases (45%), and five cases of progressive diseases (25%). Median overall survival was 39 months (Figure 4), and median progression-free survival was 9.5 months (Figure 5).

These outcomes should be interpreted descriptively only, given the absence of a control group and the exploratory nature of the study.

Survival and tumor response outcomes are presented descriptively and should not be interpreted as evidence of a causal effect of the ketogenic diet.

### 3.5. Exploratory Analysis of Tumor Biology

Four patients underwent cytoreductive nephrectomy after receiving immune checkpoint inhibitor therapy combined with the ketogenic diet. For each of these patients, PD-L1 expression was assessed in the pretreatment tumor biopsy and compared with expression on the nephrectomy specimen. A reduction in PD-L1 expression after treatment and KD was observed in three of the four tumors analyzed, while one patient showed no significant change (Table 5). Given the limited sample size and the absence of a control group, these findings should be considered exploratory and hypothesis-generating only. Variations in PD-L1 expression may reflect treatment effects, temporal and spatial tumor heterogeneity, sampling variability between biopsy and nephrectomy specimens, tumor evolution over time, or the effect of the ketogenic diet.

## 4. Discussion

Recent advances in systemic therapy have markedly improved outcomes for patients with metastatic renal cell carcinoma (mRCC). Modern first-line combinations of immune checkpoint inhibitors (ICIs) with tyrosine kinase inhibitors (TKIs), or dual ICI therapies, now achieve median overall survival estimates approaching or exceeding 50 months in selected populations, increasing 3-year survival rates in recent cohorts [2]. Despite these improvements, mRCC remains incurable for most patients, and disease progression ultimately occurs. Interest therefore persists in complementary strategies targeting tumor biology, including metabolic interventions such as the ketogenic diet (KD).

Patient recruitment in the CETOREIN trial was rapid, with only one patient declining to participate, reflecting strong patient interest in integrating dietary strategies with systemic therapy. However, this pilot study highlights the challenge of maintaining long-term adherence to KD in this context. Only 40% of patients completed the intended 12-month intervention. Systemic therapy–related side effects—such as anorexia, nausea, and fatigue, affecting up to 70% of patients treated with ICI [20]—likely compounded the burden of a restrictive diet. Based on our experience, a planned duration of six months appears more realistic than one year and may represent an optimal compromise between feasibility and metabolic impact.

The strict 2:1 lipid-to-protein+carbohydrate ratio also proved challenging. Many patients required an adjustment to a 1.5:1 ratio to maintain adequate protein intake, especially those with obesity or baseline malnutrition. This observation is consistent with the recognized difficulty of implementing KD in patients with cancer-related anorexia or cachexia. KD, therefore, appears less suitable for patients who are severely malnourished or at high nutritional risk.

We observed a modest initial weight loss during the first month, followed by stabilization or regain, consistent with the expected metabolic adaptation phase and with other observations coming from ketogenic interventions in other diseases [22,23]. Importantly, patient-reported food diaries showed good concordance with ketonuria measurements, supporting the reliability of dietary self-monitoring in this population.

Medium-chain triglyceride (MCT) supplementation was implemented to facilitate ketosis induction; however, the BetaQuik^®^ formulation likely contributed to gastrointestinal side effects, including diarrhea, gastric discomfort, and nausea. These symptoms often improved once MCT intake was reduced or discontinued. For future studies, careful selection of MCT formulations and dosing strategies will be essential to minimize gastrointestinal side effects.

Overall, KD was well tolerated, and no severe diet-related adverse events were observed. We observed increases in cholesterol and creatinine levels during follow-up, which should be interpreted cautiously. These changes may partially reflect expected metabolic effects of ketogenic interventions, transient dehydration, dietary lipid intake, concomitant systemic therapies, or attrition-related selection effects over time. Concerns regarding cardiovascular risk associated with KD have been raised, but recent analyses report no significant association with cardiovascular mortality [24].

Our findings are consistent with previous studies evaluating KD in other solid tumors. Pilot trials in glioblastoma have reported acceptable safety but limited long-term adherence due to the restrictive nature of KD and gastrointestinal intolerance [12]. Small trials in breast and prostate cancer confirmed good tolerability, transient weight loss, and metabolic improvements, without demonstrating clear antitumor activity [8]. In pancreatic cancer, adherence was particularly poor in cachectic patients, reinforcing our observation that KD is not suitable for individuals with significant malnutrition [25]. Collectively, these data align with our experience in metastatic RCC: KD is feasible and has demonstrated an acceptable tolerability profile in selected patients under close nutritional monitoring, despite frequent gastrointestinal adverse events, but adherence remains challenging, and no robust efficacy signal has been demonstrated across cancer types.

Regarding clinical outcomes, we did not observe any signal of enhanced antitumor efficacy. Treatment responses and survival kinetics were consistent with expectations for patients receiving contemporary systemic therapies. Given the small sample size, the absence of randomization, and the lack of a control arm, no conclusions regarding the efficacy can be drawn.

In the exploratory biological analysis, PD-L1 expression decreased in three of four tumors in patients who underwent cytoreductive nephrectomy after KD combined with immunotherapy, compared to baseline biopsy and surgical specimens. Although intriguing, this observation is based on very limited numbers and should be interpreted with caution. The observed changes cannot be attributed to the ketogenic diet and may instead reflect immunotherapy effects, temporal tumor evolution, or spatial heterogeneity between biopsy and nephrectomy specimens.

Although ketogenic diets have been hypothesized to influence tumor metabolism, inflammatory signaling, and immune modulation through ketone-body–mediated immunometabolic pathways, their implementation in patients with advanced cancer raises important practical and nutritional considerations. In particular, β-hydroxybutyrate may interact with inflammatory and immune pathways beyond its role as an alternative energetic substrate [18]. However, in metastatic cancer patients receiving systemic therapy, the preservation of nutritional status, maintenance of lean body mass, long-term tolerability, and prevention of treatment-related malnutrition remain critical priorities.

Interpretation of safety and clinical outcomes is limited by the heterogeneity of the study population, including differences in treatment regimens, treatment lines, histological subtypes, and disease burden. In addition, the small sample size, non-randomized design, absence of a control group, and patient attrition further limit conclusions regarding clinical efficacy.

## 5. Conclusions

The CETOREIN pilot study demonstrates that a ketogenic diet is feasible and safe for selected patients with metastatic renal cell carcinoma, although long-term adherence remains challenging. A 12-month intervention was difficult to sustain, and our findings suggest that shorter durations—approximately six months—and more flexible macronutrient ratios may improve compliance. Gastrointestinal intolerance, likely related in part to MCT supplementation, was the main limitation but did not result in severe toxicity. Early weight loss was modest and transient, and metabolic monitoring showed good concordance between dietary records and ketonuria.

No meaningful signal of enhanced antitumor efficacy was observed, as expected in a small, non-randomized feasibility study. Nevertheless, the acceptable safety profile and strong patient interest support further investigation. Future trials should incorporate optimized dietary protocols, improved nutritional support, and more tolerable MCT formulations.

Larger, prospective, randomized studies are now required to determine whether metabolic modulation through a ketogenic diet can meaningfully influence clinical outcomes in metastatic renal cell carcinoma.

## Figures and Tables

**Table 1:** Baseline and clinical characteristics for patients receiving ketogenic diet—CETOREIN study.

Characteristic	*n*
Men, *n* (%)	17 (80.95)
Mean age	61.6 (37–78)
Mean BMI	27.7 (19–43.6)
BMI > 30, *n* (%)	5 (23.8)
ECOG at screening, *n* (%)	
0	17 (81)
1	4 (19)
Histology, *n* (%)	
RCC	17 (81)
Papillary	1 (4.8)
Chromophobe	1 (4.8)
Unclassified high-grade renal cell carcinoma	1 (4.8)
Xp11 translocation renal cell carcinoma	1 (4.8)
T stage, *n* (%)	
1	1 (4.8)
2	3 (14.3)
3	15 (71.2)
4	2 (9.5)
ISUP grade, *n* (%)	
2	5 (23.8)
3	12 (57.1)
4	2 (9.5)
Previous nephrectomy, *n* (%)	13 (61.9)
Metastases sites, *n* (%)	
Lung	17 (81)
Lymph node	9 (42.9)
Adrenal	5 (23.8)
Liver	5 (23.8)
Bone	5 (23.8)
Other	5 (23.8)
IMDC risk groups, *n* (%)	
Favorable	5 (23.8)
Intermediate	10 (47.6)
Poor	6 (28.6)
Metastatic treatment line, *n* (%)	
First-line treatment	18 (85.7)
Other lines	3 (14.3)
mRCC treatment, *n* (%)	
NIVOLUMAB + IPILIMUMAB	13 (61.9)
AXITINIB + PEMBROLIZUMAB	4 (19)
NIVOLUMAB	1 (4.8)
AXITINIB	2 (9.5)
CABOZANTINIB	1 (4.8)

**Table 2:** Composition of diet before and during protocol according to food diary—CETOREIN study. Components of diets used are listed in grams per day.

Component (Mean g/Day)	Before Study (*n* = 20)	KD 1 Month (*n* = 15)	KD 3 Months (*n* = 11)	KD 6 Months (*n* = 10)	KD 12 Months (*n* = 8)
Fat	92.1	166.3	187.3	188.6	186.0
Carbohydrates	174.8	40.8	53.4	54.7	69.1
Protein	80.9	86.1	93.9	100.2	103.9
Energy (kCal)	1854	2060	2274	2317	2366
Ketogenic ratio	NA	1.5	1.5	1.4	1.2

The ketogenic ratio was calculated according to the following formula: fats/(protein + carbohydrates).

**Table 3:** Clinical and laboratory parameters during the study—CETOREIN study.

Parameters	Before Study *n* = 21	KD 1 Month *n* = 19	KD 3 Months *n* = 15	KD 6 Months *n* = 11	KD 12 Months *n* = 8
Weight (kg) (mean ± SD)	83.6 (12.2)	82 (12.8)	81.2 (16.2)	74.9 (9.5)	78.3 (8)
Mean weight difference (%) (SD)	NA	−2.1 (2)	8.1 (16.2)	0.3 (6.1)	0.2 (7.3)
Albumin (g/L) (mean ± SD)	39.2 (2.9)	39.6 (3.2)	35.8 (3.8)	41.2 (2.7)	39.5 (2.2)
Glucose (g/L) (mean ± SD)	0.9 (0.08)	1 (0.07)	0.9 (0.07)	0.9 (0.08)	1 (0.07)
Creatinine (µmol/L) (mean ± SD)	93.4 (14.6)	93.8 (15.96)	93.5 (14.66)	109.2 (16.65)	113.9 (10.24)
Total cholesterol (g/L) (mean ± SD)	1.7 (0.3)	2 (0.3)	2 (0.3)	2.3 (0.4)	2.5 (0.8)
Triglycerides (g/L) (mean ± SD)	1.3 (0.4)	1.9 (1.2)	1.4 (0.4)	1.3 (0.4)	1.5 (0.5)
Vitamin D (nmol/L) (mean ± SD)	65.9 (15.8)	69.3 (11.4)	68.1 (11)	85.6 (13)	88.1 (21)
Magnesium (mmol/L) (mean ± SD)	0.8 (0.04)	0.8 (0.04)	0.9 (0.05)	0.8 (0.04)	0.8 (0.02)
Selenium (µg/L) (mean ± SD)	85.6 (8.1)	89.1 (8.7)	87.9 (12.5)	99.1 (7.4)	99.5 (5.9)
Total carnitine (µmol/L) (mean ± SD)	53.8 (5.9)	61.1 (7.3)	56.8 (6.6)	49.3 (8.9)	49 (6)

**Table 4:** Adverse events possibly related to KD were reported during the study, per the CTCAE V4—CETOREIN study.

Adverse Event (CTCAE V4)	Grade 1–2, *n* (%)	Grade 3–4, *n* (%)	Overall, *n* (%)
Diarrhea	11 (55)	-	11 (55)
Weight loss	9 (45)	1 (5)	10 (50)
Hypercholesterolemia	8 (40)	-	8 (40)
Hypertriglyceridemia	7 (35)	1 (5)	8 (40)
Dyspepsia	6 (30)	-	6 (30)
Hyperuricemia	4 (20)	-	4 (20)
Hypoalbuminemia	3 (15)	-	3 (15)
Abdominal pain	3 (15)	-	3 (15)
Anorexia	2 (10)	-	2 (10)

**Table 5:** Expression of PD-L1 on biopsy before treatment and diet, and on renal tissue after exposure to ketogenic diet and treatment—CETOREIN study.

TPS (PD-L1) % Biopsy	TPS (PD-L1) % Kidney
15%	1%
80%	<1%
20%	<3%
5%	5%

## Data Availability

The data supporting the findings of this study are available upon reasonable request from the corresponding author at cyrielle.rolley@chu-angers.fr.

## Abbreviations

The following abbreviations are used in this manuscript:

KD	Ketogenic diet
ICI	Immune checkpoint inhibitors
mRCC	Metastatic renal cell carcinoma
MCT	Medium-chain triglyceride
RCC	Renal cell carcinoma
TKI	Tyrosine kinase inhibitors
